# The Circular RNA Cdr1as Promotes Myocardial Infarction by Mediating the Regulation of miR-7a on Its Target Genes Expression

**DOI:** 10.1371/journal.pone.0151753

**Published:** 2016-03-21

**Authors:** Hai-Hua Geng, Rui Li, Ya-Min Su, Jie Xiao, Min Pan, Xing-Xing Cai, Xiao-Ping Ji

**Affiliations:** 1 The Key Laboratory of Cardiovascular Remodeling and Function Research, Chinese Ministry of Education and Chinese Ministry of Health, Shandong University Qilu Hospital, Jinan, 250012, Shandong, China; 2 Department of Cardiology, Affiliated Hospital of Nantong University, Nantong, 226001, Jiangsu, China; University of Cincinnati, College of Medicine, UNITED STATES

## Abstract

**Objectives:**

Recent studies have demonstrated the role of Cdr1as (or CiRS-7), one of the well-identified circular RNAs (circRNAs), as a miR-7a/b sponge or inhibitor in brain tissues or islet cells. This study aimed to investigate the presence of Cdr1as/miR-7a pathway in cardiomyocytes, and explore the mechanism underlying the function of miR-7a in protecting against myocardial infarction (MI)-induced apoptosis.

**Methods:**

Mouse MI injury model was established and evaluated by infarct size determination. Real-time PCR was performed to quantify the expression of Cdr1as and miR-7a in cardiomyocytes. Cell apoptosis was determined by caspase-3 activity analysis and flow cytometry assays with Annexin V/PI staining. Transfection of Cdr1as overexpressing plasmid and miR-7a mimic were conducted for gain-of-function studies. Luciferase reporter assay and western blot analysis were performed to verity potential miR-7a targets.

**Results:**

Cdr1as and miR-7a were both upregulated in MI mice with increased cardiac infarct size, or cardiomyocytes under hypoxia treatment. Cdr1as overexpression in MCM cells promoted cell apoptosis, but was then reversed by miR-7a overexpression. The SP1 was identified as a new miR-7a target, in line with previously identified PARP, while miR-7a-induced decrease of cell apoptosis under hypoxia treatment was proven to be inhibited by PARP-SP1 overexpression. Moreover, Cdr1as overexpression *in vivo* increased cardiac infarct size with upregulated expression of PARP and SP1, while miR-7a overexpression reversed these changes.

**Conclusions:**

Cdr1as also functioned as a powerful miR-7a sponge in myocardial cells, and showed regulation on the protective role of miR-7a in MI injury, involving the function of miR-7a targets, PARP and SP1.

## Introduction

Myocardial infarction (MI) has been one of the leading causes of death and disability around the world due to its contribution to most mortalities associated with coronary artery disease [1]. Normally, MI can be caused by acute occlusion of the coronary artery [2]. During MI development, prolonged myocardial ischemia contributes to the myocardial cell death process, resulting in cardiomyocyte loss, after which scar formation and pathological left ventricle (LV) remodeling triggered by ischemia stress promote cardiac dysfunction, leading ultimately to heart failure [3]. Because of the terminally differentiated characteristics of cardiomyocytes, great attention has been paid to cell based therapies in repairing loss of cardiomyocytes after MI injury [4, 5]. Additionally, since apoptosis, one programmed cell death, has shown great contribution to cardiomyocyte loss during acute MI [6], efforts have also been made to explore the molecular mechanisms of apoptotic loss of cardiomyocytes [7, 8], with revealed potential therapeutic value [9]. However, the mechanism underlying cardiomyocyte apoptosis during MI injury is still not fully understood.

Circular RNA (or circRNA) is a new type of untranslated RNA with the potential to form a covalently closed continuous loop, which has been detected in various organisms [10–13], and shows abundantly expression across the eukaryotic tree of life [14] with evolutionary conservation particularly between humans and mice. Similar to linear mRNAs, the well-expressed, stable expression of circRNAs also shows tissue and developmental stage-specific characteristics [15, 16], indicating several potential biological functions. Recently, one human circRNA, antisense to the cerebellar degeneration-related protein 1 transcript (Cdr1as) [17], also termed as ciRS-7 (circular RNA sponge for miR-7) [18], has been identified to share expression domains with miR-7 [19]. In the mouse brain, particularly in the neocortical and hippocampal neurons, Cdr1as and miR-7 were observed to be co-expressed [18]. Moreover, the overexpression of Cdr1as was proven to induce developmental defects in the midbrain of embryonic zebrafish, similar to that induced by miR-7 inhibition, while miR-7 overexpression could partially rescue the Cdr1as-induced phenotype [19]. These studies supported the key role of Cdr1as as a miR-7 sponge or inhibitor by binding miR-7 seed sites. And, under different mechanisms, Cdr1as might also act as a miR-7 buffer or reservoir in modulating miR-7 function [20].

Our previous study reported the upregulated expression of miR-7a/b in murine hearts with myocardial ischemia-reperfusion (I/R) injury [21], while miR-7a/b functioned in protecting myocardial cells against I/R-induced apoptosis by negative regulation on poly(ADP-ribose) polymerase (PARP), an executioner of apoptosis [22]. Previous study also showed that miR-7a expression is upregulated by unfolded protein response (UPR) associated with MI development, and simulated *in vitro* ischemia in cardiomyoblasts [23], while ectopic expressed miR-7a provides resistance against UPR-mediated apoptosis. However, miR-7a upregulation by simulated I/R or MI showed a conflict with its protective role against stress-induced apoptosis in cardiomyocytes, indicating an unrevealed regulatory mechanism needed to be clarified. Therefore, in this study, Cdr1as was speculated to be co-expressed with miR-7a in cardiomyocytes after MI injury. Further functional studies were performed to illustrate the potential mechanism of the Cdr1as/miR-7a pathway during MI-induced apoptosis, including the exploration of new miR-7a target genes related to the apoptotic process.

## Materials and Methods

### Mouse MI Model

Male C57BL/6 mice of 8-week-old were housed with free access to chow and water under standard conditions and subjected to an alternated 12 h light/dark cycle before experiments. Mice were randomly divided into two groups (*n* = 10 for each) and anesthetized with sodium pentobarbital (50 mg/kg) by intraperitoneal injection to receive MI introduction or a sham operation under aseptic conditions. MI introduction was performed by permanent ligation of the left anterior descending (LAD) artery as previously described [7]. Mice receiving a sham operation were subjected to the same surgical procedures as the experimental ones, but the LAD artery was not ligated. After MI or sham surgery, the health conditions of mice were monitored on a daily basis with the assistance of veterinary staffs and no unexpected deaths were observed in this study. The pain or distress of mice was also monitored to ensure the analgesic effect as described [24]. At indicated time points, mice were euthanized by the intracardial injection of cardioplegic solution to arrest the heart at diastolic phase for following removal of heart tissues and further analysis. All animal experiments were performed in accordance with to the Animal Management Rules of the Chinese Ministry of Health (document No. 55, 2001) and approved by the Animal Care Committee of Shandong University.

### Determination of Myocardial Infarct Size

At 24 h post-ligation, the mice were anesthetized and Evans blue (2%; Sigma-Aldrich, Raleigh, NC, USA) was injected into the aorta to stain all myocardial tissues except the area at risk (AAR), as ischemic tissues. The left ventricle (LV) was isolated and transversely cut into1 mm thick slices. Then, LV slices were stained with 1% triphenyltetrazolium chloride (TTC; Sigma-Aldrich) for 15 min at 37°C to distinguish ischemic and infarcted tissues. The areas of infarction without staining were designated as non-viable, whereas non-infarcted areas with blue staining were identified as viable. The AAR and the infarct area were determined separately, and the infarct size was expressed by a percentage of AAR as described before [25].

### Lactate Dehydrogenase (LDH) Release Assay

Before mice were sacrificed, blood samples were collected for serum preparation, and LDH concentration in the serum was measured by using a colorimetric LDH assay kit (Nanjing Jiancheng, Jiangsu, China) according to the manufacture’s protocol.

### Quantitative Real-Time Polymerase Chain Reaction (RT-PCR)

The TRIzol reagent (Invitrogen, Carlsbad, CA, USA) was used to isolate total RNA, including microRNA, according to the manufacturer’s instructions. For mRNA expression, cDNA synthesis was performed with Oligo (dT), and a SYBR RT-PCR kit (Takara, Otsu, Japan) was used for mRNA quantification with specific primers. For the detection of Cdr1as expression, specific primers were used (Forward: 5’-GTGTCTCCAGTGTATCGGCG-3’; Reverse: 5’-TACTGGCACCACTGGAAACC-3’) as described before [26]. For miR-7a expression, microRNA was analyzed by using the TaqMan MicroRNA Reverse Transcription Kit (Applied Biosystems, Foster City, CA, USA) with provided RT-U6 and microRNA-specific stem-loop primers, and the expression levels were determined through TaqMan MicroRNA assays with the TaqMan Universal PCR Master Mix (Applied Biosystems). All reactions were preceded on the ABI 7500 real-time PCR system (Applied Biosystems) by standard protocols. The comparative threshold cycle (Ct) value was calculated and analyzed by using the 2^-ΔΔCT^ method, with U6 and β-actin as an internal control.

### Cell Culture

Primary cardiomyocytes, isolated from mouse ventricles as previously described [27], and mouse cardiac myocytes (MCM) cell line, obtained from the American Type Culture Collection (ATTC, Manassas, VA, USA), were cultured in Dulbecco's Modified Eagle Medium (DMEM, Invitrogen-Gibco) containing 10% FBS and 100 μg/ml penicillin/streptomycin at 37°C under 5% CO_2_. For hypoxia treatment, cells were incubated in a hypoxic chamber for 24 h.

### Plasmids and microRNAs

Plasmid for the co-expression of PARP and SP1 (pCDNA-PARP-SP1) was constructed by Genechem (Shanghai, China) via inserting a splicing junction of mouse PARP and SP1 sequence into a pCDNA3.1 vector (Invitrogen, USA). Cdr1as expression plasmid (pCDNA-Cdr1as) containing a *Cdr1as* sequence with invert repeat flanking introns for circular *Cdr1as* production and its scrambled sequence plasmid with no circular product were kindly provided by Dr. T. Hansen (Aarhus University, Denmark). Lentiviral vectors carried miR-7a mimic (miR-7a mimic) and its scrambled control microRNA (Pre-NC) were obtained from Genepharma (Shanghai, China), as well as corresponding lentivirus (1×10^9^ TU/ml) for in vivo studies.

### Transfection

For cell transfection, MCM cells were pre-cultured overnight until 50% confluence and then transfected with plasmids or microRNAs by Lipofectamine 2000 (Invitrogen) following the manufacturer’s instructions. At 6h post transfection, the supernatant of cell cultures was removed and fresh medium was added. After incubation of an additional 48 h, cells were collected for further experiments. For *in vivo* analysis, plasmids pre-treated with Lipofectamine 2000 or lentivirus prepared by Genepharma, were delivered by intramyocardial injection into three sites of the left ventricular free wall with an insulin syringe (BD Biosciences, San Jose, CA, USA). After injection, mice were allowed to recover for three days, and then sham or MI surgery was performed.

### Annexin V/Propidium Iodide (PI) Staining

Myocardial cell apoptosis was assayed by using an FITC-labeled Annexin V (Annexin V-FITC) apoptosis detection kit (BD Biosciences). Cells were collected after the indicated treatment, washed twice with cold PBS and then resuspended in 1×binding buffer, followed by staining with Annexin V-FITC and PI in the darkness. Immediately, the percentage of apoptotic cells was quantified by flow cytometry (FACSCalibur, BD Biosciences) according to the manufacturer’s instructions.

### Caspase-3 Activity Analysis

Caspase-3 activity was measured by a Colorimetric Assay Kit (KeyGen, China). Cells were lysed, and the supernatant was collected for protein concentration assay with the BCA method. Samples containing equal amounts of protein were incubated with 2×reaction buffer and specific substrate at 37°C for 4 h in the dark. Absorbance at 405 nm was measured on a microplate reader (HynergyTM HT, BIOTEK, Winooski, VT, USA), and results were normalized to those from control cells.

### Luciferase Assay

The 3’ untranslated region (UTR) sequence of SP1 or PARP containing the predicted mo-miR-7a binding sites was amplified from MCM genome and sub cloned into the multiple cloning sites in the downstream of the luciferase gene in the GV126 vector (Promega Corporation, Durham, NC, USA), resulting in the wild-type luciferase reporter construct, GV126-SP1-3’UTR-WT or GV126-PARP-3’UTR-WT. The mutant types, GV126-SP1-3’UTR-MU and GV126-PARP-3’UTR-MU, with the corresponding mutant *seed* sequence, were synthesized by site-directed mutagenesis [28]. These GV126 constructs expressing firefly luciferase and pRL-cmv vectors expressing *Renilla* luciferase (Genechem) were co-transfected into cells using Lipofectamine 2000 (Invitrogen). Luciferase activity was measured with the Dual-Luciferase Reporter Assay System (Promega Corporation), with *Renilla* luciferase activity as an internal control.

### Western Blot Analysis

Whole lysates were prepared from cultured cardiomyocytes, and protein concentration was determined by the BCA method. Samples with equal amounts of protein were separated by SDS-PAGE and transferred to PVDF membranes (Millipore, Bedford, MA, USA) for the detection of PARP, SP1 and GAPDH or β-actin levels after being incubated with specific antibodies from Cell Signaling Technology (Danvers, Massachusetts, USA). Immunoreactive bands were visualized on the ImageQuant LAS 4000 System (GE Healthcare, Wisconsin, USA) by using an enhanced chemiluminescence kit (Millipore).

### Immunohistochemistry

Immunohistochemical staining was performed for the detection of SP1 and PARP expression in the myocardium. Briefly, partial mouse hearts, removed 24 h after the ligation, were fixed with 4% PFA in PBS, embedded in paraffin and routinely processed for frozen sectioning into 4μm slices. Then, sections were deparaffinized and subjected to antigen retrieval by heating in citrate buffer and stained with specific antibodies for SP1 or PARP. After being counter-stained with hematoxylin, images of sections were acquired with an image analysis system (GE Healthcare) and evaluated with the Image Pro Plus 6.0 software (Media Cybernetics, Baltimore, MD, USA). The mean optical density of SP1 or PARP was determined by calculation of the ratio of integrated optical density in the area of interest.

### Statistical Analysis

All data were expressed as the mean ± SD. Statistical significance of the means between two independent groups was analyzed by Student’s *t*-test (two-tailed) or one-way analysis of variation (ANOVA), while two-way ANOVA was used for comparisons among multiple groups, with *P* < 0.05 considered as statistically significant.

## Results

### Co-Expression of Cdr1as and miR-7a Was Detected during MI-Induced Injury or in Hypoxia-Treated Cardiomyocytes

**Fig 1:**
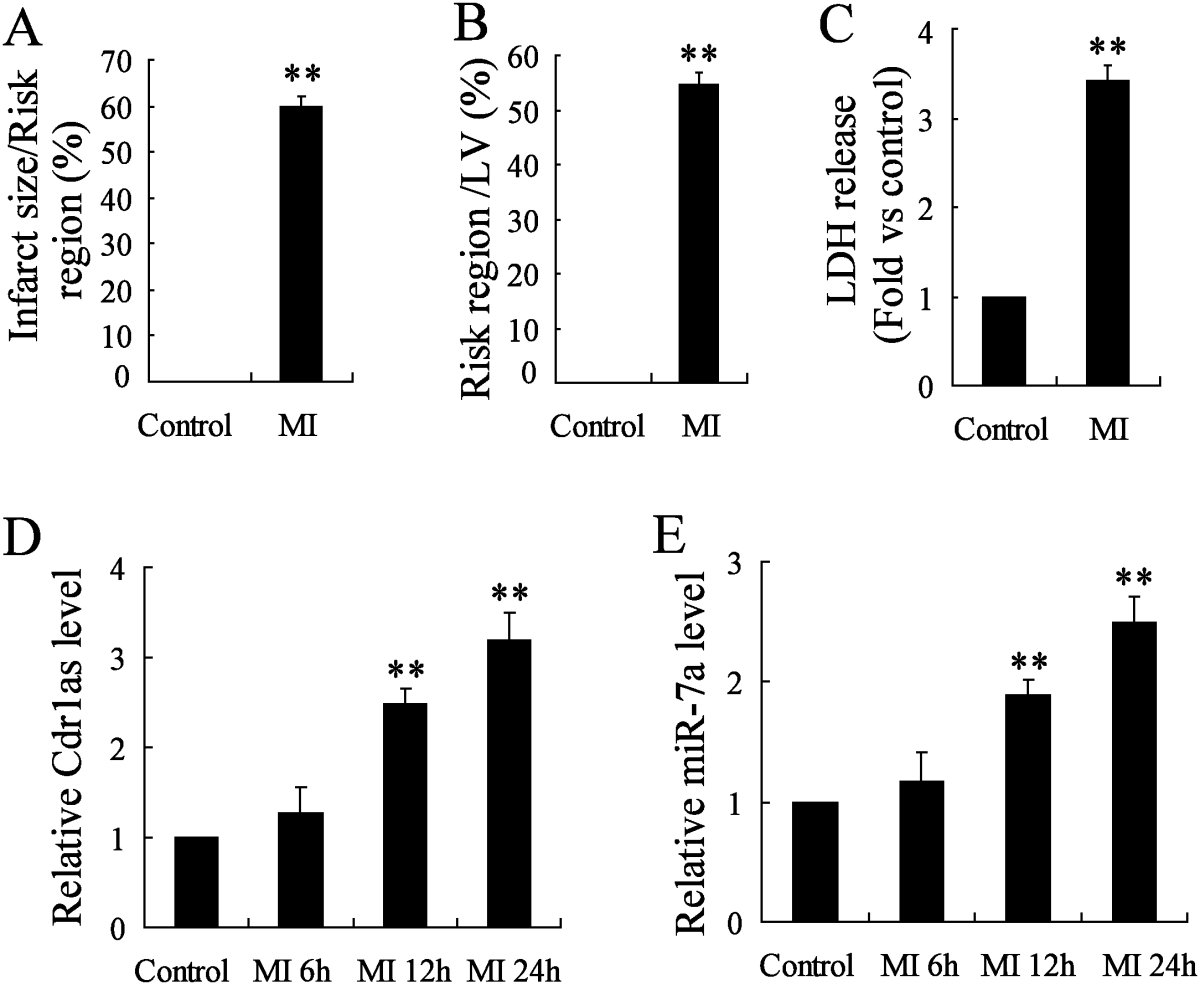
Upregulated expression of Cdr1as and miR-7a was detected in myocardial tissues from MI mice. The infarct size (A), area at risk (B), and lactate dehydrogenase (LDH) release (C) were analyzed to examine the established mouse MI model, along with a sham group (control). And, myocardial tissues were disassociated at 6, 12, or 24 h after MI introduction, to detect the expression of Cdr1as (D) and miR-7a (E) by qRT-PCR analysis. *n* = 6, **P < 0.01 vs. control.

**Fig 2:**
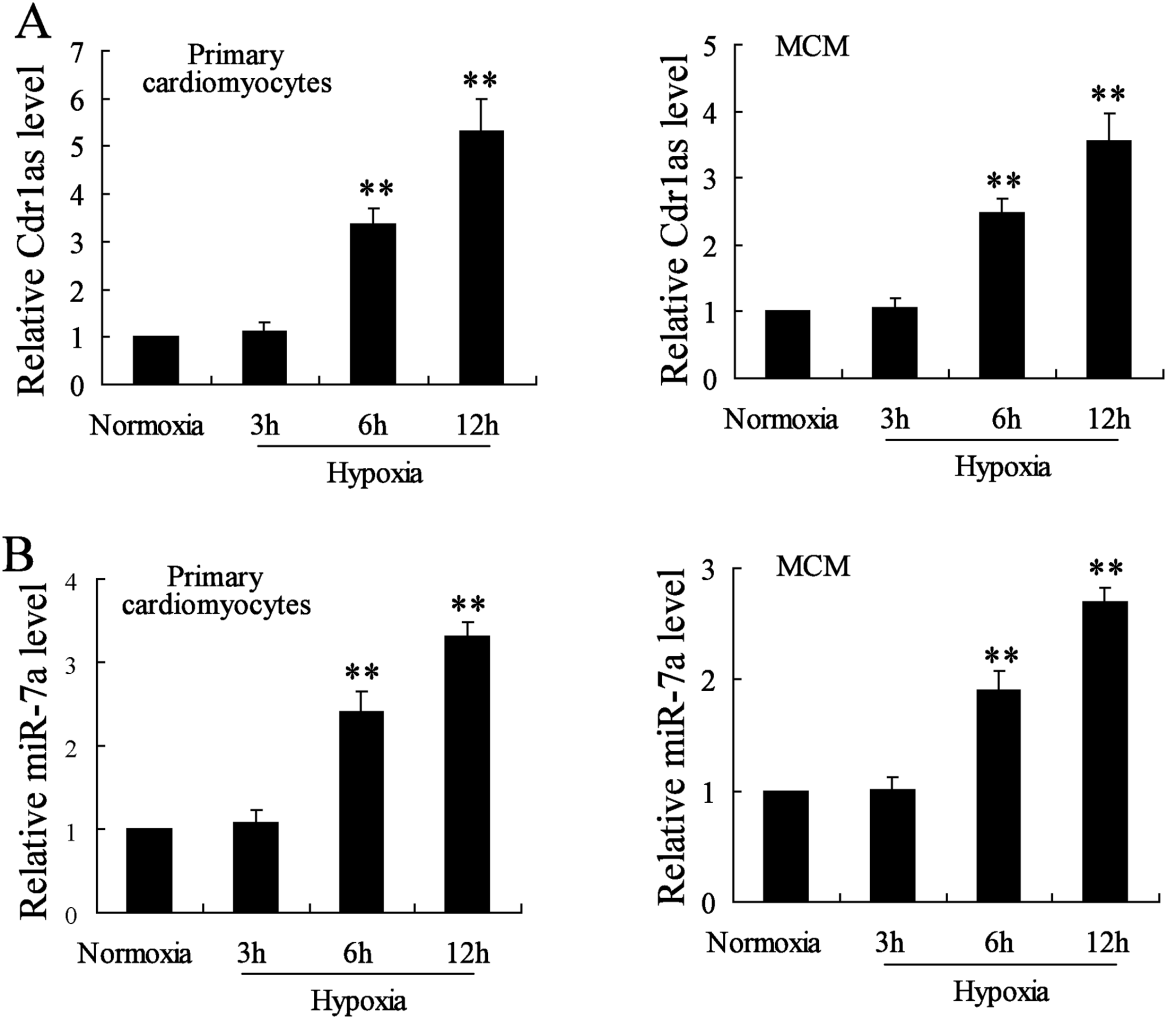
Cdr1as expression was elevated in hypoxia-treated cardiomyocytes with overexpressed miR-7a. Primary cardiomyocytes, obtained from C57BL/6 mice, and mouse myocardia cell line MCM were subjected to hypoxia treatment for 3, 6, or 12 h for RNA expression analysis by qRT-PCR. (A) Expression of Cdr1as. (B) Expression of miR-7a. *n* = 3, **P < 0.01: compared to cells under normoxia condition.

Since the potential role of Cdr1as as a miR-7 sponge has been revealed in the mouse brain [18], it was hypothesized that Cdr1as may also be co-overexpressed with miR-7a in cardiomyocytes during MI-induced injury. To evaluate the MI development in mice, myocardial infarct size and the extent of AAR were detected accordingly, as well as LDH release, a biochemical marker of myocardial cell necrosis [29]. A significant increase of infarct size, AAR and LDH release were observed in MI mice as compared with the sham control (*P* < 0.01; Fig 1A–1C), indicating the successful establishment of a mouse MI model. Additionally, the expression of Cdr1as and miR-7a showed a respective 2.4-fold and 1.9-fold increase in cardiomyocytes at 24 h after MI surgery (*P* < 0.01; Fig 1D and 1E). Moreover, the co-expression of Cdr1as and miR-7a was also confirmed in both primary cardiomyocytes from normal CL57B6 mice and MCM cells with a time dependent manner after hypoxia treatment for simulating MI injury *in vitro* (Fig 2). These results indicated the presence of a Cdr1as/miR-7a pathway in murine cardiomyocytes wherein the potential function of miR-7a in regulating cardiomyocyte apoptosis might also be mediated by Cdr1as.

### Overexpression of miR-7a Protected Cardiomyocyte from Cdr1as-Induced Apoptosis

**Fig 3:**
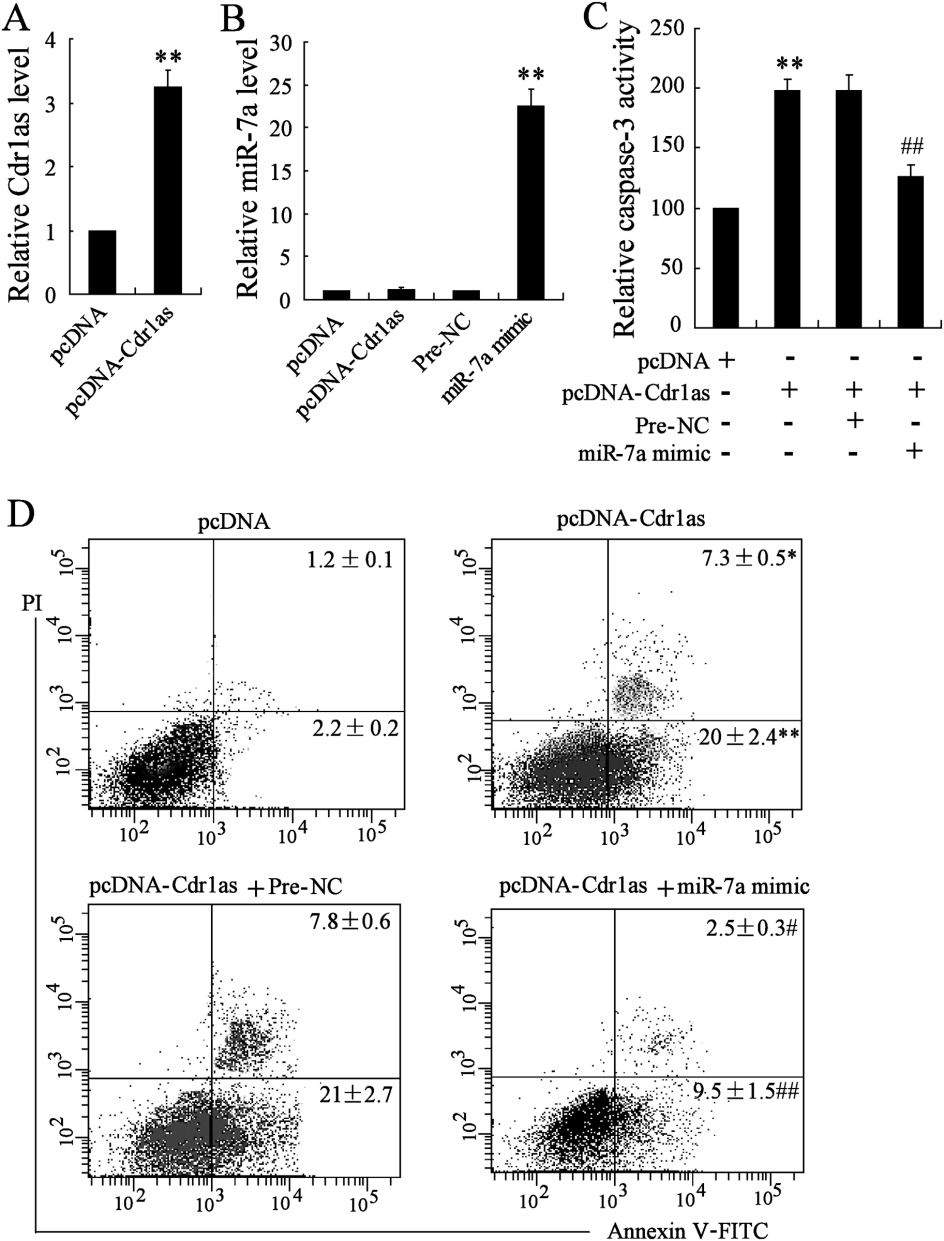
MiR-7a overexpression reversed Cdr1as-induced apoptosis of MCM cells. MCM cells were transfected with pcDNA-Cdr1as or miR-7a mimic for the overexpression of Cdr1as and miR-7a respectively, with pcDNA or Pre-NC as the respective negative control. After the transfection, Cdr1as or miR-7a expression level was confirmed by qRT-PCR. Analysis for caspase-3 activity and cell apoptosis were performed by the colorimetric method and Annexin V/PI staining respectively. A. Expression of Cdr1as. B. Expression of miR-7a. C. Relative caspase-3 activity under different conditions. D. Representative flow cytometry of apoptosis of MCM cells. The percentage of apoptotic cells with positive Annexin V signal were analyzed and compared accordingly. *n* = 3, *P < 0.05, **P<0.01: compared to cells transfected with pcDNA (A, C, D) or Pre-NC (B) as control; ^#^*P* < 0.05, ^##^*P* < 0.01: compared to cells co-transfected with pcDNA-Cdr1as and Pre-NC.

Given the fact that miR-7 overexpression shows regulation on stress-induced apoptosis of cardiomyocytes [21, 23], it is possible that ectopic Cdr1as expression may also influence cardiomyocyte apoptosis due to their co-expression. Firstly, Cdr1as overexpression plasmid (pcDNA-Cdr1as) or miR-7a mimic was transfected into MCM cells for 48h, and Cdr1as or miR-7a expression was confirmed to be increased by around 3.3-fold or ~22-fold respectively, as compared to the control (Fig 3A and 3B), while the expression levels of endogenous miR-7a showed no obvious changes under pcDNA-Cdr1as transfection (Fig 3B). Then, the caspase-3 activity, one common used detector for apoptosis, and the percentage of apoptotic cells were analyzed in cells with Cdr1as overexpression or co-overexpression of Cdr1as and miR-7a. As shown in Fig 3C and 3D, Cdr1as significantly increased the caspase-3 activity and apoptotic cells, while co-overexpression of miR-7a reversed Cdr1as-induced phenotypes, resulting in reduced caspase-3 activity and apoptotic cells. These results implied that the Cdr1as overexpression might regulate cardiomyocyte apoptosis by reducing miR-7a activity, due to its regulation on apoptosis-related targets.

### MiR-7a Targeted the Expression of PARP and SP1, which Were Upregulated by Cdr1as

**Fig 4:**
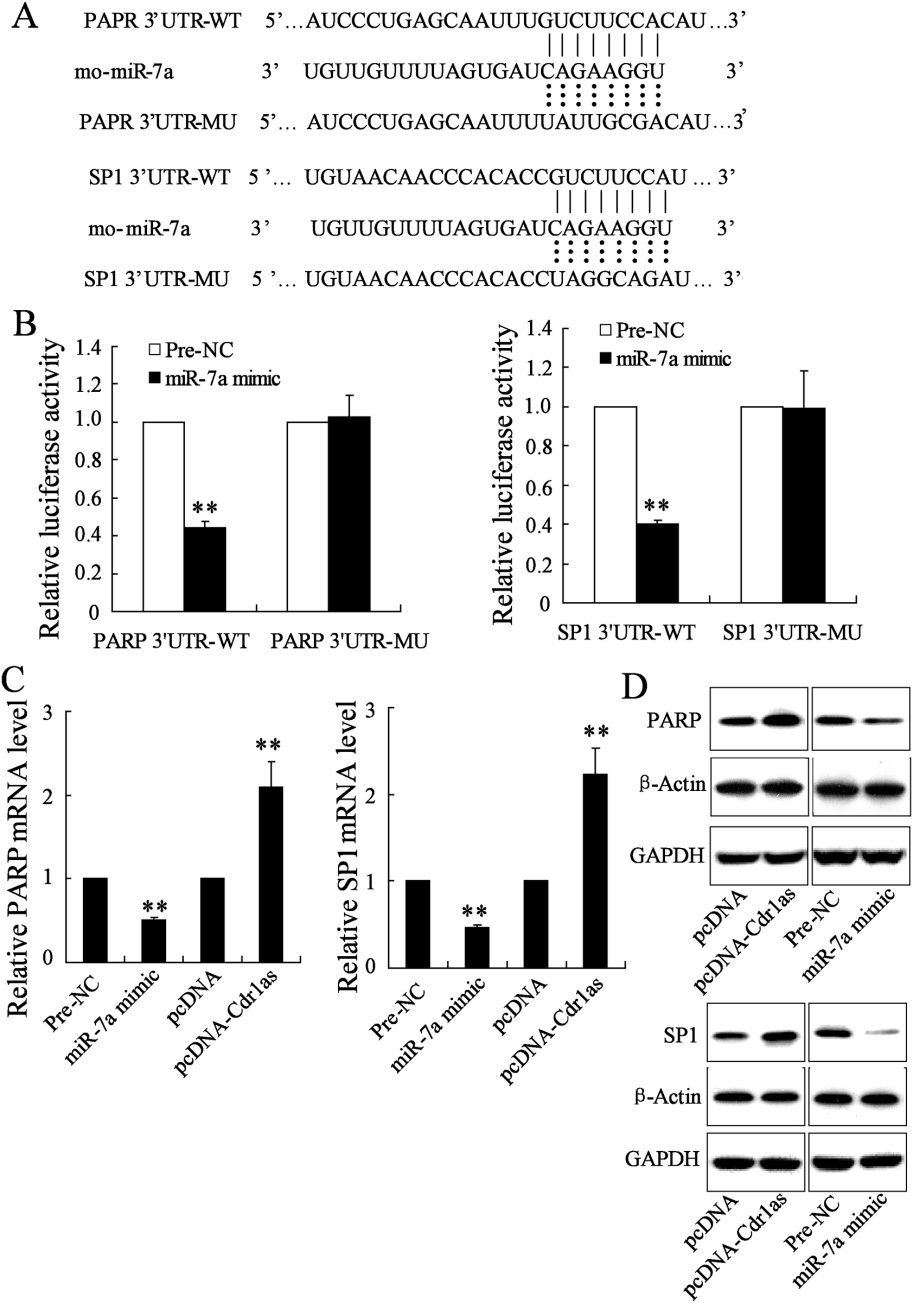
Regulation of miR-7a on the expression of PARP and SP1 was inhibited by Cdr1as. A. Conserved miR-7a binding sites in 3’-UTR of PARP and SP1. WT: wild type; MU: mutant type. B. Luciferase activity of the 3’-UTR of PARP and SP1 in miR-7a overexpressing cells, after normalized to that of *Renilla*. Cells transfected with Pre-NC were used as control. C. RT-PCR analysis of mRNA level of PARP and SP1 under miR-7a or Cdr1as overexpression. Cells transfected with Pre-NC (for miR-7a mimic) or pcDNA (for pcDNA-Cdr1as) were used as control D. Western blot analysis of protein level of PARP and SP1. *n* = 3, **P < 0.01 vs. control.

To elucidate the molecular mechanisms of miR-7a in cardiomyocyte apoptosis after MI injury, putative targets involving the apoptotic program were screened by using TargetScan, one microRNA target prediction program (http://www.targetscan.org/). SP1, one transcription factor implicated in hypoxic gene transcription [30], was found to behave conserved binding sites for miR-7a (Fig 4A). To confirm whether miR-7a targeted SP1 through its 3’UTR, a firefly luciferase reporter plasmid containing the 3’UTR segment of SP1 mRNA with the putative miR-7a binding sites was constructed (GV126-SP1 3’UTR-WT), while another mutant luciferase reporter vector containing the mutated binding sites (GV126-SP1 3’UTR-MU) was used as negative control. After the transfection of miR-7a mimic or negative control microRNA (Pre-NC) into MCM cells, luciferase reporters were co-transfected for 48 h. Results showed that miR-7a strongly inhibited luciferase activity of the GV126-SP1 3’UTR -WT, but not the GV126-SP1 3’UTR -MU (*P* < 0.01, Fig 4B), indicating its direct target on SP1. Similar analysis for PARP, another miR-7a target that has been demonstrated to function in I/R-induced apoptosis in our previous study [21], was also performed here for further investigation (Fig 4A and 4B). In addition, the expression of PARP and SP1 at both mRNA and protein level were detected to be inhibited significantly in miR-7a overexpressing cells (Fig 4C and 4D). In contrast, however, the expression of both two target genes was strongly increased by the overexpression of Cdr1as through the transfection of pcDNA-Cdr1as (Fig 4C and 4D). Thus, it could be speculated that although miR-7a expression was induced after MI injury, the synergistical increase of Cdr1as level could reduce miR-7a activity by sponging miR-7a, leading to the upregulated expression of miR-7a targets, including PARP and SP1, which play pro-apoptotic roles during MI development.

### Overexpression of miR-7a Protected MCM Cells from Hypoxia-Induced Cell Apoptosis by Targeting PARP and SP1

**Fig 5:**
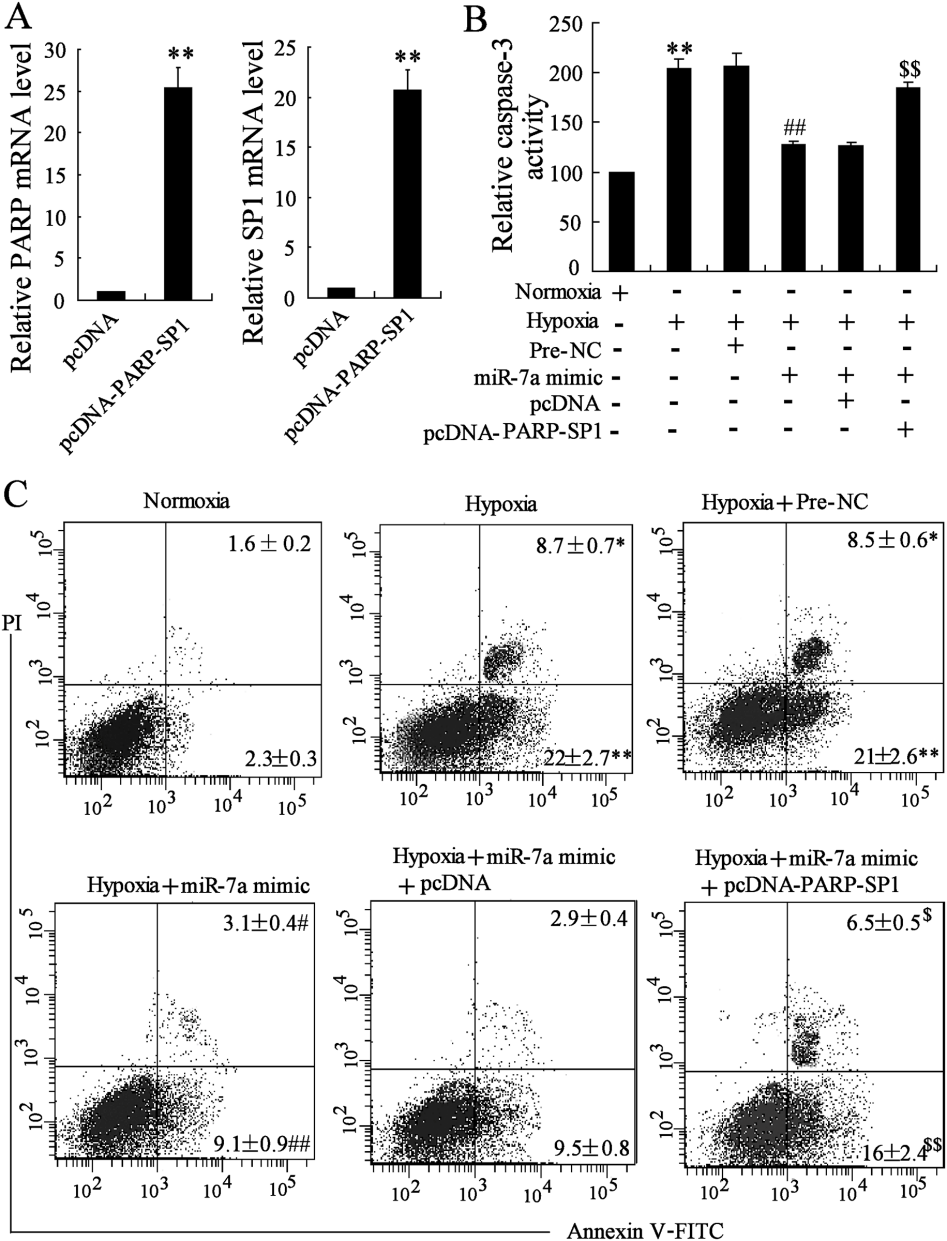
Hypoxia-induced apoptosis was mediated by the targeting of miR-7a on PARP and SP1. MCM cells were transfected miR-7a mimic or co-transfected with miR-7a mimic and pcDNA-PARP-SP1, and then subjected to hypoxia treatment. A. Expression of PARP. B. Expression of SP1. C. Relative caspase-3 activity under different conditions. D. Representative flow cytometry of apoptosis of MCM cells. PI: propidium iodide. *n* = 3, *P < 0.05, **P < 0.01: compared to cells with pcDNA transfection (A, B) or under normoxia condition (C, D); ^#^*P* < 0.05, ^##^*P* < 0.01: compared to cells with Pre-NC transfection under hypoxia condition; ^$^*P* < 0.05, ^$ $^*P* < 0.01: compared to cells co-transfected with miR-7a mimic and pcDNA under hypoxia condition.

To verify whether miR-7a and its targets, PARP and SP1, potentially functioned in the apoptotic process during MI injury, miR-7a overexpressing MCM cells were subjected to hypoxia treatment, and cell apoptosis with activated caspase-3 was analyzed accordingly (Fig 5C), as well as the percentage of apoptotic cells (Fig 5D). Hypoxia treatment induced significant activation of caspase-3 with increased apoptotic cells, while the overexpression of miR-7a could rescue these apoptosis phenotypes induced by hypoxia. Additionally, pcDNA-PARP-SP1 was transfected into MCM cells, and the expression levels of PARP and SP1 were confirmed to be both upregulated (Fig 5A and 5B), suggesting the co-overexpression of PARP and SP1. Further results showed that hypoxia-induced apoptosis could be recovered by the co-overexpression of PARP and SP1, through the co-transfection of pcDNA-PARP-SP1 into miR-7a overexpressing cells (Fig 5C and 5D). These results illustrated that miR-7a protected cardiomyocytes from hypoxia-induced apoptosis by inhibiting the function of its targets, PARP and SP1.

### Overexpression of Cdr1as Aggravated MI Injuries with Upregulated PARP and SP1, which Could Be Rescued by the Co-Overexpression of miR-7a

**Fig 6:**
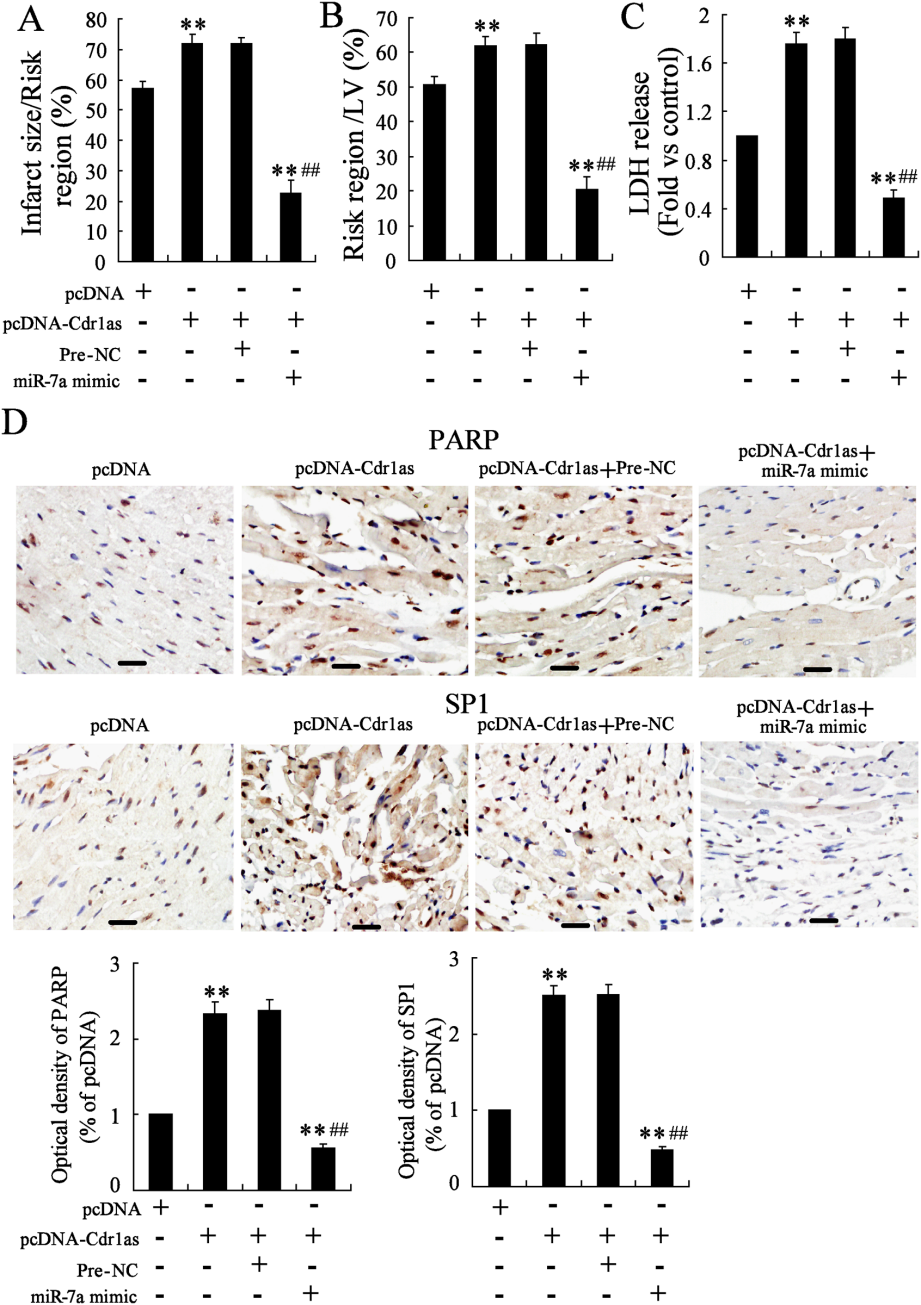
MiR-7a overexpression reversed Cdr1as-induced bad effects in mouse MI model. Three days after the transfection of pcDNA or pcDNA-Cdr1as, mice were subjected to MI introduction. Effect of Cdr1as overexpression on MI-induced infarct size (A), area at risk (B), lactate dehydrogenase (LDH) release (C), and the expression of PARP and SP1 (D; bar = 100μm) was analyzed, as well as miR-7a co-overexpression with Cdr1as through the delivery of miR-7a mimic three days before MI introduction. *n* = 6, **P < 0.01: compared to MI mice with pcDNA transfection; ^##^*P* < 0.01: compared to MI mice co-transfected with pcDNA-Cdr1as and Pre-NC.

Since Cdr1as acted as an apoptosis inducer in cardiomyocytes, and was also proven to induce the upregulation of PARP and SP1 probably by sponging miR-7a *in vitro*, the overexpression of Cdr1as was performed *in vivo* through the transfection of pcDNA-Cdr1as via intracardial injection to verify whether Cdr1as functioned through upregulating PARP and SP1 in MI mice. As expected, MI injuries, including enlarged infarct size, increased AAR and elevated LDH release, were all strongly aggravated by Cdr1as overexpression (Fig 6A–6C), while the expression of PARP and SP1 in heart tissues were upregulated consistently (Fig 6D). Nevertheless, Cdr1as-induced effects could all be reversed by the overexpression of miR-7a through the co-transfection of lentivirus with miR-7a mimic (Fig 6), supporting that miR-7a could protect cardiomyocytes from Cdr1as-induced aggravation of MI injuries, involving the re-downregulated expression of PARP and SP1. Therefore, the circular RNA Cdr1as also functioned as a miR-7a sponge in promoting MI injuries by reduced the activity of miR-7a in inhibiting the expression of its target genes, PARP and SP1. Moreover, miR-7a showed potential therapeutic value for improving MI-related injuries.

## Discussion

The presence of circRNAs has just been identified to be widespread and substantial in the eukaryotic tree of life until very recently [14], but only a few of them have been well-characterized and functionally demonstrated to be microRNA sponges or inhibitors [15, 16]. The circular RNA Cdr1as is derived from an antisense transcript of the CDR1 protein-coding gene at chromosome X (NC_000086.7) in mice [17], containing binding sites or clusters for binding miR-7, and function as a miR-7 sponge in neuronal cells [18], or in newly reported islet cells [26]. However, the potential role of Cdr1as in myocardial cells has not yet been explored although miR-7a has been reported to be overexpressed in stress-stimulated cardiomyocytes [21, 23]. Thus, in the present study, the presence of the Cdr1as/miR-7a pathway was characterized by detecting their co-expression in cardiomyocytes with MI induction, to explore whether Cdr1as showed similar negative effects on miR-7a function in cardiomyocytes.

The mouse myocardial infraction model was successfully established with observed enlargement of infarct size, increase of AAR and LDH release, and the expression of Cdr1as and miR-7a were both detected to be significantly upregulated in myocardial tissues of MI mice as compared to the control. Similarly, co-upregulated Cdr1as and miR-7a were confirmed in hypoxia-treated cardiomyocytes, as simulation of MI *in vitro*. These results illustrated that Cdr1as indeed co-expressed with miR-7a in myocardial cells. According to previous studies, the co-expression of Cdr1as and miR-7a indicate potential interaction between them, wherein Cdr1as can function as a miR-7 inhibitor, buffer or reservoir under different conditions by sponging miR-7, showing regulation on miR-7 function [20]. Thus, in order to analyze the potential effects of Cdr1as on miR-7a function, the overexpression of Cdr1as and miR-7a was performed successively in a murine myocardial cell line (MCM). Results showed that Cdr1as-induced apoptosis, with increased caspase-3 activity, could be reversed by miR-7a co-overexpression. Additionally, the expression levels of endogenous miR-7a were detected to be not induced by Cdr1as overexpression, while miR-7a overexpression alone also showed little effect on the apoptosis of normal cultured cardiomyocytes (data not shown), in line with our previous studies [21]. Thus, it was suggested that miR-7a might only function in regulating stress-induced apoptosis of myocardial cells. These results also implied that miR-7a activity, in protecting against cardiomyocytes apoptosis as indicated previously [21, 23], was inhibited by Cdr1as overexpression.

The pivotal role of microRNAs has also been reported in heart diseases involving the degradation or translational inhibition of their target mRNAs [31]. For example, miR-24 inhibits cardiomyocyte apoptosis by regulating its target gene Bim, one B-cell lymphoma 2 (Bcl2) family members, in a mouse MI model [7]. MiR-34a expression in circulation was increased in MI conditions, during which it could promote cardiomyocyte apoptosis via negatively regulating aldehyde dehydrogenase 2 (ALDH2) [8], an anti-apoptotic enzyme with declined activity upon myocardial injury [32, 33]. Our previous study also demonstrated that PARP is a functional target gene of miR-7a/b involved in miR-7a/b—mediated cardiomyocyte protection against I/R in a rat model [21]. In the current study, the predicted mo-miR-7a binding sites at the 3’ UTR of PARP were also observed through bioinformatics analysis, while further luciferase assays and expression detection under miR-7a overexpression demonstrated it to be one conserved miR-7a target.

Notably, SP1, a member of the SP/KLF family of transcription factors [34], was also identified as one target gene of miR-7a. A mechanism for SP1-regulated induction of cardiac CD39 has been reported in tissue protection during myocardial ischemia [30]. Recently, it has also been proposed that SP1 might play crucial regulatory roles in MI development, including cardiac fibrosis, apoptosis and angiogenesis, due to its close association with MI-related miRNAs [35]. For example, SP1 expression can be inhibited by miR-21-5p [36], which leads to reduced activation of SP1 targets, including fas ligand (FASLG) and vascular endothelial growth factor A (VEGFA) [37, 38], resulting in inhibiting apoptosis and angiogenesis. In addition, miR-29b-3p, one miR-29 family member, can regulate cardiac fibrosis by modulating collagen type I alpha 1 (COL1A1) either directly or indirectly through SP1 [39], since SP1 can also regulate the expression of COL4A1 [40]. In this study, SP1 was also validated as a new miR-7a target in cardiomyocytes through luciferase assays and gain-of-function studies. Therefore, it can be speculated that SP1 also functions in miR-7a mediated cardiomyocyte apoptosis during MI, in line with PARP.

In order to verify this speculation, the effects of miR-7a and its targets, PARP and SP1, on hypoxia-induced apoptosis were analyzed in cardiomyocytes under the overexpression of miR-7a and the subsequent co-overexpression of PARP and SP1. Results showed that hypoxia-induced apoptosis was inhibited by miR-7a, but was then reversed by the co-overexpression of PARP and SP1. Besides, hypoxia-induced apoptosis could also be reversed by the transfection of PARP or SP1 siRNA (data not shown), similar to the transfection of miR-7a mimic, indicating that miR-7a protected cardiomyocytes from hypoxia-induced cell apoptosis by targeting PARP and SP1. Indeed, the upregulation of SP1 and PARP was also observed in Cdr1as overexpressing cardiomyocytes, indicating the role of Cdr1as, as one important regulator to prevent miR-7a from interacting with its target transcripts. In addition, Cdr1as overexpression in vivo could aggravate MI development with increased cardiac infract size, as well as strongly upregulated PARP and SP1, while miR-7a overexpression significantly attenuated these Cdr1as-induced changes.

In conclusion, this study illustrated the presence of the Cdr1as/miR-7a axis in cardiomyocytes for the first time. Further results revealed the mechanism of the Cdr1as/miR-7a pathway in MI injury, wherein Cdr1as acted as a miR-7a sponge by inhibiting the regulation of miR-7a on its targets. These observations explain why upregulated miR-7a showed cardiomyocyte protection during MI injury. It is important to note that SP1 was proven to be a new functional target of miR-7a involved in miR-7a mediated cardiomyocytes protection against MI injury. Further studies could focus on the pathophysiological role of Cdr1as for new therapeutic strategies in MI treatment.
